# Subtle Cryptococcal Immune Reconstitution Inflammatory Syndrome

**DOI:** 10.7759/cureus.6138

**Published:** 2019-11-12

**Authors:** Ranjan K Singh

**Affiliations:** 1 Internal Medicine, Anti-Retroviral Therapy Centre, District Hospital, Khagaria, IND

**Keywords:** cryptococcal infection, hiv, iris

## Abstract

Patients with HIV and opportunistic infections (OIs), such as tuberculosis and cryptococcosis, have been recognised to have immune reconstitution inflammatory syndrome (IRIS) following initiation of highly active antiretroviral therapy (HAART). The initiation of HAART boosts CD4 T cells, which result in exacerbations of OIs. We have identified deterioration of neural lesion in imaging after the initiation of HAART in a patient with HIV/cryptococcal infection, although patient's clinical conditions have not deteriorated. It is evident that IRIS is in subtlety. This has been possible because of prompt treatment with antifungal drugs for the cryptococcal infection, followed by early initiation of HAART.

## Introduction

Patients with HIV and opportunistic infections, such as tuberculosis and cryptococcosis, have been recognised to have immune reconstitution inflammatory syndrome following initiation of highly active antiretroviral therapy (HAART). The neurological manifestations following HAART may be devastating. Cryptococcal meningitis-immune reconstitution inflammatory syndrome (IRIS) reportedly has an incidence rate of 13%-30% in patients with HIV coinfections [1]. It may occur either by the unmasking of a subclinical cryptococcal infection or, paradoxically, by the worsening of a pre-existing cryptococcal infection following HAART. Various factors predispose patients to paradoxical IRIS [2]. These factors may be low inflammatory response, low baseline CD4+ T cell count and high cryptococcal antigen load in cerebrospinal fluid (CSF). Manifestation of IRIS ranges from self-limiting to fulminating [3], but rapid fungicidal induction followed by prompt initiation of HAART reduces exacerbation of IRIS. In this study, we have identified IRIS in subtlety in a HAART-naive HIV patient presenting with subacute dementia. 

## Case presentation

**Figure 1:**
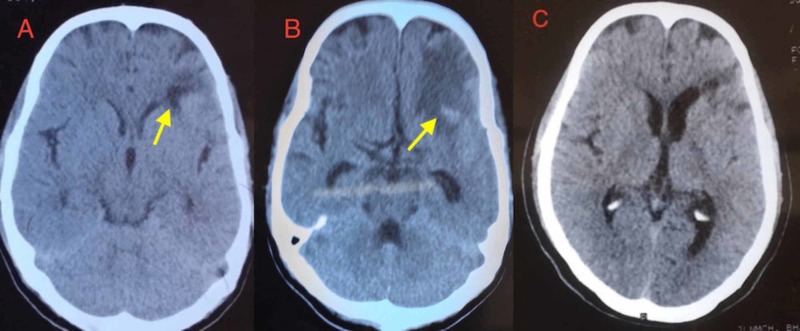
CT scans of the brain Panel A showing hypodense lesion in the left frontal lobe.Panel B showing hypodense lesion with elements of peripheral enhancement in the left frontal lobe. Panel C showing resolution of lesion.

A 32-year-old HAART-naive HIV seropositive male weighing 48 kg presented with forgetfulness, irritability and incontinence of urine, along with fever and headache for the last two months. His CD4+ cell counts were 36/μl, and he was seronegative for hepatitis B and C. He did not experience seizure or took anti-epileptic medication. Clinical examination revealed neurocognitive impairment with a mini-mental status score of 14/30. There was no focal neurological deficit. A computed tomography (CT) of the brain (Figure 1A) showed a hypodense lesion in the left frontal lobe. CSF analysis confirmed cryptococcal meningitis with protein 27 mg/dl (ref value:15-45 mg/dl), sugar 29 mg/dl (ref. value>55 mg/dl), cells 32/cmm (lymphocytes) and Cryptococcus Antigen Latex Agglutination System 4+. The rapid CSF Cryptococcus Antigen Latex Agglutination assay was the preferred diagnostic approach. The treatment regimen of amphotericin B in the dose of 0.7 mg/kg of body weight in 5% dextrose IV infusion daily for 12 days and fluconazole (400 mg) orally (in place of flucytosine) per day was initiated. Serum electrolytes were monitored during amphotericin B infusion. Antiretroviral drugs such as tenofovir, lamivudine and efavirenz were started after two weeks of treatment with antifungal drugs. Fluconazole 400 mg/day was continued for 10 weeks. Following the treatment, neurocognitive functions improved markedly with a mini-mental state score of 26/30 and CD+ T-cell counts of 165/μl. However, CT image (Figure 1B) still showed a heterogeneous hypodense lesion with elements of peripheral enhancement in the left frontal lobe.

Fluconazole (200 mg per day) was continued along with HAART. At the end of six months, CD4+ cell counts were up to 246/μl and a further CT image (Figure 1C) showed a resolution of the neurolesion.

## Discussion

The hypodense lesion (Figure 1A) is the result of the perivascular accumulation of yeast (cryptococcosis) and its mucinous product. These lesions can be found on convexities, in addition to their predilection for other sites in the brain [4]. After eight weeks of HAART, CT brain (Figure 1B) shows signs of inflammation, and in fact, leptomeningeal enhancement is not possible without HAART [5]. The eight-week antiretroviral treatment has led to improvement in both clinical status and neurocognitive function. The patient’s CD4+ T-cell counts have increased from 36/μl to 165/μl; however, there is a simultaneous worsening of the neurolesion in CT imaging as evidenced in Figure 1B compared to Figure 1A. This result is suggestive of paradoxical IRIS in subtlety and has occurred due to rapid initiation of antifungal drugs and prompt initiation of HAART.

Cattelan et al. [6] have reported new lesions appearing in neuroimages following HAART in two patients of cryptococcal meningitis with HIV infections. These patients do not have any positive findings in neuroimages prior to HAART. 

In a retrospective study between 2005 and 2014, the imaging pattern in patients receiving HAART is found to be different from neuroimages in patients without HAART. All the patients on HAART show leptomeningeal and parenchymal enhancement in cortical and subcortical region of brain under immune reconstitution, while those without HAART have pseudocysts and lacunar infarcts [7].

## Conclusions

About one-third cases of cryptococcal meningitis experience mild to devastating form of IRIS after the initiation of HAART. In our case, neuroimaging enhancement in the left frontal lobe of the brain without any clinical exacerbation following HAART has been observed. This subtle form of IRIS is the result of rapid treatment of cryptococcal infection with antifungal drugs and prompt initiation of HAART.

## References

[1] Sungkanuparph S, Filler SG, Chetchotisakd P (2009). Cryptococcal immune reconstitution inflammatory syndrome after antiretroviral therapy in AIDS patient with cryptococcal meningitis: a prospective multicenter study. Clin Infect Dis.

[2] Longley N, Harrison TS, Jarvis JN (2013). Cryptococcal immune reconstitution inflammatory syndrome. Curr Opin Infect Dis.

[3] Johnson T, Nath A (2010). Neurological complications of immune reconstitution in HIV-infected populations. Ann N Y Acad Sci.

[4] Walot I, Miller BL, Chang L, Mehringer CM (1996). Neuroimaging findings in patients with AIDS. Clin Infect Dis.

[5] Mathews VP, Alo PL, Glass JD, Kumar AJ, McArthur JC (1992). AIDS-related CNS cryptococcosis: radiologic-pathologic correlation. AJNR Am J Neuroradiol.

[6] Cattelan AM, Trevenzoli M, Sasset L, Lanzafame M, Marchioro U, Meneghetti F (2004). Multiple cerebral cryptococcomas associated with immune reconstitution in HIV-1 infection. AIDS.

[7] Katchanov J, Branding G, Jefferys L, Arastel K, Stocker H, Sibert E (2016). Neuroimaging of HIV-associated cryptococcal meningitis: comparison of magnetic resonance imaging findings in patients with and without immune reconstitution. Int J STD AIDS.

